# Measure and spatial identification of social vulnerability, exposure and risk to natural hazards in Japan using open data

**DOI:** 10.1038/s41598-023-27831-w

**Published:** 2023-01-12

**Authors:** Theo Raduszynski, Muneyoshi Numada

**Affiliations:** Department of Civil Engineering, The University of Tokyo, Tokyo, Japan

**Keywords:** Climate-change impacts, Climate-change mitigation, Sustainability, Natural hazards, Environmental impact

## Abstract

Understanding the location of risk to natural hazards, namely the areas of high exposure and vulnerability is a major priority that was identified by the Sendai framework for Disaster Reduction 2015–2030 in order to reach substantial reduction of disaster risk. It is also a necessary decision-making tool for disaster mitigation policy-makers in Japan and around the world. This paper successfully develops a simple methodology using only open data to build the first large-scale (whole country), fine resolution (sub-municipal level) social vulnerability analysis in the context of five different types of natural hazards (earthquake, tsunami, storm surge, flooding and landslide). The result is then compared to an indicator of exposure of population to these hazards in Japan in order to propose a representation of disaster risk. Results show that vulnerability in Japan is highly heterogeneous with urban/rural and north/south fractures. Combining the social vulnerability index with exposure analysis, results show a wide variety of spatial patterns of risk areas in Japan.

## Introduction

We can understand the risk to a given natural hazard as a function of the hazard (as magnitude and probability of occurrence), the exposure (of elements and people) and the vulnerability (of exposed elements and people)^1^. While hazards can be described as processes of defined magnitudes and frequencies, the exposure of populations is grasped through spatial analysis of human settlements in areas affected by such hazards. The last aspect, related to the vulnerability of the people, is in some of its dimensions linked to the observation that some populations such as the poor, elderly or disables are generally facing greater consequences of disasters induced by natural hazards than the general public. Although these different social groups may have a similar degree of exposure to a given natural hazard, it will have varying effect on each group, since they bear different capabilities. The term *social vulnerability* is used to describe this phenomenon among its other dimensions, this concept being very specific to a given context and geographical situation, as we will explain in the following paragraphs.

First, we will provide definitions for the terms hazard, disaster, exposure, vulnerability and risk based on the *2009 UNISDR Terminology on Disaster Risk Reduction*. A hazard is *a process, phenomenon or human activity that may cause loss of life, injury or other health impacts, property damage, social and economic disruption or environmental degradation*. A disaster is *a serious disruption of the functioning of a community or a society at any scale due to hazardous events interacting with conditions of exposure, vulnerability and capacity, leading to human, economic or environmental losses and impacts*. The exposure to natural hazards is defined as *the situation of people, infrastructure, housing, production capacities located in hazard-prone areas*. It is added that measures of exposure can include the number of people or types of assets in an area. These can be combined with the specific vulnerability and capacity of the exposed elements to any particular hazard to estimate the quantitative risks associated with that hazard in the area of interest. The UNISDR defines vulnerability as *the conditions determined by physical, social, economic and environmental factors or processes which increase the susceptibility of an individual, a community, assets or systems to the impacts of hazards*. It is also added that the term capacity refers to the positive factors which increase the ability of people to cope with hazards. Risk is defined as *the combination of the probability of an event and its negative consequences*. Many approaches understand risk as a combination of hazard, exposure, and vulnerability^1^. For instance, the European Union Index for Risk Management INFORM is a composite indicator developed as a tool for understanding the risk of humanitarian crisis and disasters, combining 54 indicators into three dimensions of risk: hazards and exposure to them, vulnerability and thelack of coping capacity.

Social vulnerability is defined as *the set of characteristics of a group or individual in terms of their capacity to anticipate, cope with, resist and recover from the impact of a natural hazard. It involves a combination of factors that determine the degree to which someone’s life and livelihood is at risk by a discrete and identifiable event in nature or society*^2^. The idea that impacts of natural hazards on population are not only determined by the hazard characteristics and the exposure of human settlements but also the adjustments and characteristics of this exposed population has been discussed as early as 1945^3^. It later inspired attempts to measure vulnerability, with a classic, data-driven approach to develop social vulnerability indices established by Cutter et al. in 2003^4^, who proposed the construction a social vulnerability index of U.S. countries using factor analysis. It has then been applied and adapted in the context of many different countries and types of hazard, such as Australia^5^, China^6^, Germany^7^, Japan^8^^,^^9^, Nepal^10^ and many more.

It is also important to consider other dimensions of vulnerability than social vulnerability. Birkmann^11^ identifies key components of vulnerability as: social, economic (propensity for loss of economic value from damage), physical (potential for damage to physical assets), cultural (potential for damage to intangible values including meanings placed on artefacts, customs, habitual practices and landscapes), environmental (potential for damage to all ecological and bio-physical systems) and institutional (potential for damage to governance systems, organizational form and function). In 2019, Papathoma-Khöle^12^ presented a Physical Vulnerability Index for buildings exposed to dynamic flooding in mountain areas, considering indicators such as exposure, building sizes and materials, and orientation, among others. Papathoma-Khöle also presented in 2021^13^ presented a framework defining institutional vulnerability and detailing its links with other components of vulnerability, proposing indicators for its assessment such as participation of the populations, risk awareness, accountability, political stability, among others.

Recently, there has been a conceptual evolution regarding social vulnerability indices, which at first were mostly limited to the characteristics of individuals, to considering their relation to the society in terms of support capabilities. This evolution and approach is exemplified in Portugal^14^ where they distinguished, in social vulnerability, the *criticality* related to the characteristics of individuals and communities (social assistance, employment, demography, economic dynamics, education, housing and health for example) and the *support capabilities* which are related to systems or infrastructures that are aimed at supporting the individuals (the economy, or the structure of buildings for example). From this perspective, we can see that social vulnerability indicators often include broader dimensions of the vulnerability mentioned in the previous paragraph, such as physical or economic vulnerabilities.

In 2015, the United Nations organized Sendai framework for Disaster Reduction 2015–2030, setting 4 priorities for action in order to reach “substantial reduction of disaster risk and losses in lives”. The first of these priorities, *Understanding disaster risk* is described as follows:

Policies and practices for disaster risk management should be based on an understanding of disaster risk in all its dimensions of vulnerability, capacity, exposure of persons and assets, hazard characteristics and the environment. Such knowledge can be leveraged for the purpose of pre-disaster risk assessment, for prevention and mitigation and for the development and implementation of appropriate preparedness and effective response to disasters.

In this context, expanding approaches using publicly available data in order to deepen knowledge on vulnerability and exposure of populations to hazards is very much needed. Japan is well-known for the frequency and variety of its disasters induced by natural hazards, and Japanese national and local governments have made available a large amount of data on predicted exposure and magnitude of various hazards. For instance, after the 2011 Great East Japan earthquake and tsunami, the Law on Tsunami Disaster Prevention and Community Development (Law No. 123 of 2011) requires prefectures to provide tsunami scenario and associated flooding assumptions. The results are available on the Ministry of Land, Infrastructure and Transport open data website^15^. Prefecture-level aggregated data on hazard zones for various type of hazards are also available. Also, most Japanese municipalities provide hazard maps^16^ that show the estimated extent and magnitude, but almost never give precise information on the exposed population and the areas of socially vulnerable populations.

Consequently, social vulnerability indices in Japan need to be further studied and expanded. Some approaches have focused on precise regions or cities, such as Liangxiao^8^ who developed a vulnerability index for Tokyo Katsushika Ward. Conversely, Fraser^9^ proposed social vulnerability and capital indices from publicly available data for each of Japan’s 1741 municipalities, making possible to compare municipalities in term of overall community resilience. However, social characteristics of populations inside a given municipality can be heterogeneous. Moreover, these studies did not compare the obtained social vulnerability indexes with hazard and exposure data. Therefore, the notion of risk was not directly tackled.

In order to prioritize investments in disaster mitigation measures and urban planning, policy-makers require decision-making tools that indicate where is the most urgent need, namely the location of the most risky zones, where the exposure is high and the socially vulnerable people less likely to be able to cope and recover on their own.

*To address this deficit, this paper develops a simple methodology using only publicly available data from various sources to create the first large-scale (whole country), fine resolution (sub-municipal level) social vulnerability index of populations in Japanin the context of five different types of natural hazards (earthquake, tsunami, storm surge, flooding and landslide).* The result is then compared to an indicator of exposure to these hazards in Japan in order to propose a representation of disaster risk. This framework can be replicated in other regions of the world if similar data is available. These measures that emerge from public data can help policy makers to evaluate where it is most appropriate to invest in disaster mitigation measures (decision-making) and provide insights about the spatiality of vulnerability and risk in Japan.

## Methods

**Figure 1:**
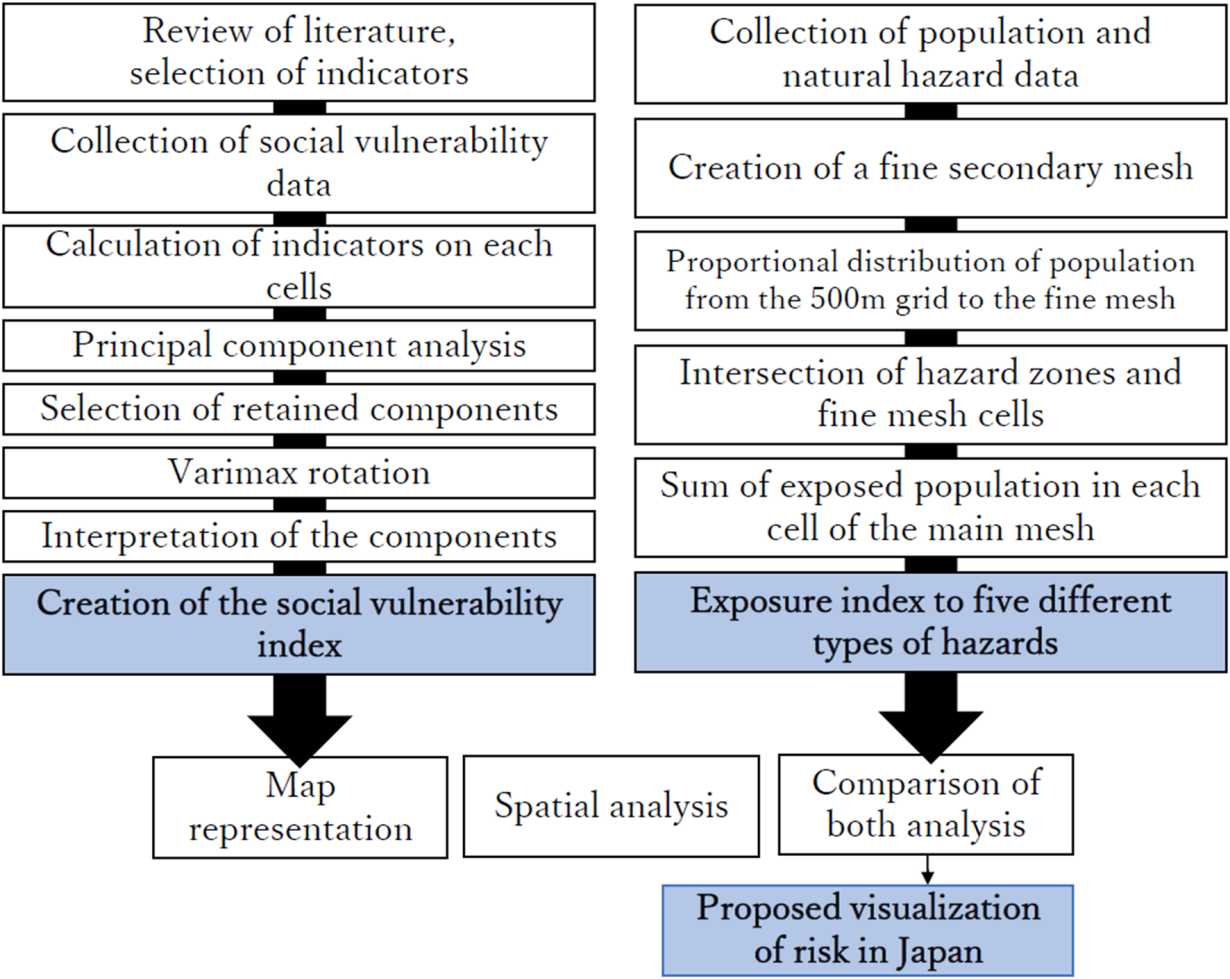
Analytical framework of this study. Data treatments, principal component analysis, Varimax rotation and exposure analysis are detailed in this “Methods” section.

### Natural hazards considered in this study

In this study, we decided to consider 5 types of natural hazards, as they induce more than 94% of fatalities due to disasters in the country (period: 1994–2013, White paper on disaster management, 2014, Cabinet Office, Government of Japan). Those hazards are earthquakes, tsunamis, storm surges, river flooding and landslides.

For earthquakes, the national seismic hazard maps are prepared by the Headquarters for Earthquake Research Promotion (HERP) which is a governmental organization. The probabilistic seismic hazard map that we have used is prepared *by calculating the probability that a given site will experience ground motion intensity exceeding a certain value within a target period. Evaluation is conducted by using a probabilistic approach on the site of occurrence, probability of occurrence, and magnitude of all earthquakes that could occur in and around Japan *(from Japan Seismic Hazard Information Station). The data is available as polygon data in a 250 × 250 m mesh all across Japan. For this study, we designated as exposed the zones where at least 6 + Japan Meteorological agency scale shaking in the next 30 years with more than 6% of probability.

For tsunamis, the national land numerical data download service compiles flooding scenario in the form of polygon data elaborated by each prefecture of Japan, as provided by the law no. 123 of 2011 on the creation of tsunami hazard prone areas. Each prefectures has to elaborate worst-case scenarios and simulate the outcome regarding inundated areas, and publicly release the results. For this study, we designated as exposed the zones where tsunami flooding of any height is expected according to those prefecture-level scenarios.

For storm surges, river flooding and landslides, it is similar to the previous paragraph, except the laws that charges prefecture to elaborate the scenarios are different. In each case, the data is available as precise polygon data files. For this study, we always considered as exposed the zones where flooding or landslide is expected according to those scenarios.

Overall, the data that we are using to modelize the hazard zones in Japan come from official sources, and take into account different scenarios of magnitudes and probability. Each scenarios are different for each prefecture of Japan providing the data, with respect to the local context.

**Table 1:** The five natural hazards considered in the study with data sources and the areas considered as exposed.

Natural hazard	Data source	Area considered as exposed zone
Earthquake	Japan seismic hazard information station	Area predicted by Japan meteorological agency to have at least 6% probability to shake intensity at least 6 + within 30 years
Tsunami	National land numerical data download service	Inside of flooding zone (prefecture level tsunami scenario)
Storm surge	National land numerical data download service	Inside of flooding zone (prefecture level storm surge scenario)
River flooding	National land numerical data download service	Inside of flooding zone (prefecture level flooding scenario)
Landslide	National land numerical data download service	Inside of landslide danger or vigilance zone (according to prefecture level designated landslide zones)

### Construction of the exposure analysis

The spatial granularity mesh chosen for this study (for exposure as well as the social vulnerability analysis) is the small area mesh (*shōchīki* in japanese), which are an official Japanese national census unit and statistical block. Their size vary depending on the population: they can be very small in dense urban zone and large in low populated countryside areas. They match with existing political units in Japan (can be a part of the totality of a district, a subunit of the municipality). Here, about 120,000 small zones in Japan are considered, covering the country land.

In this analysis, the exposure for each natural hazard was measured as the density of exposed population in each small zone. Thus, the goal is to count the exposed population to each selected natural hazard (see list in Table 1) in every small area cell (the same that are used for the social vulnerability analysis, for comparison purposes). In order to determine the exposed population, we created a secondary mesh, more precise, that is the result of the intersection of the small zone mesh with a 500 m square population grid mesh. The secondary mesh was generated using the Pairwise intersection tool of ArcGIS. In each square grid, the population was distributed on the cells of the secondary mesh proportionally to their surface. It means that this approximation is never larger than a 500 × 500 m grid cell. Then, for each natural hazard, cells of the secondary mesh were considered as exposed on the condition that the cell intersected with a high hazard intensity zone. Exposed zone were defined at the beginning of the Methods section. Then, inside each small zone cells, the population of exposed secondary mesh cells that are inside were aggregated, so we obtained the exposed population in each cell of the small zone mesh, for each natural hazard. The last step was to divide by the small zone area (computed with the Calculate geometry attributes tool of ArcGIS) to obtain the density of exposed population in each small zone for each natural hazard.

### Construction of the vulnerability index

As showed in Fig. 1, the social vulnerability index is obtained after the choice and collection of relevant indicators, and the performing of principal component analysis, which is *a statistical technique that takes as its input a matrix of interrelated socioeconomic variables, and creates a set of principal components that extract the important variations in the underlying input while reducing the noise and redundancy in the data*^17^. Then, a Varimax rotation with Kaiser normalisation is conducted to the selected components in order to ease their interpretation by rotating the axes of the components perpendicular to each other. This step will generate loadings on the different socioeconomic variables that will be taken into account for weighting in the final social vulnerability index. Following their interpretation, each component will manually be assigned a direction (plus or minus sign) whether its overall effect is assumed to increase of decrease social vulnerability.

The choice of the indicators in a social vulnerability index is indeed a crucial step, as this will have a large impact on the final output. We decided to follow the general recommendation to consider less variables (13, see Table 2 for the full list of indicators), but in which there is a strong confidence in their capacity to represent some aspects of social vulnerability^17^. We decided to consider 13 different indicators extracted from available open-data and that we consider better fitting the local situation with respect of the following criteria:

- *Supported by literature*: The indicator was used in past research in several reference studies or meta studies^18^.
- *Availability of data*: Recent quality data of this indicator exists and is usable for Japan at whole country scale with a high spatial granularity level.
- *Overall balance of the indicator pool*: As a whole, the selected pool of indicators should represent various aspect of vulnerability in a balanced and diversified way taking into account aspects of criticality related to the characteristics of individuals and communities and the support capabilities which are related to systems or infrastructures that are aimed at supporting the individuals.

As a result, we have selected the following indicators, as detailed in Table 2. *Demographic indicators* (number 1 to 6) aim to represent how many individuals need to be taken care of in case of disaster. Gender and age structure show those that require special consideration. Evolution of population in 2050 is considered as depopulation is a societal phenomenon in Japan that have implications in terms of the vulnerability of populations^19^. *Economic indicators* (number 6 to 9) include home value and its evolution, which play a positive role in measuring social status, resources and possession of insurances, as well as average income and part of renting households^20^.

*Facility and accessibility indicators* (number 10 to 13) include retail and business density, indicating to accessibility to living resources and commercial dynamism of the area. Distance to closest medical facility and shelters gives information on the remoteness of the area and the difficulty to access these essential services in time of disaster.

Links to indicator data sources are provided in the Data availability section.

Note: some indicators only exist at the municipal level. In order to use those variables to the small areas unit, our thinking was the following. Regarding real estate prices, we assumed that using the average price inside this municipality was an acceptable approximation (same value for each small area in the municipality), even if we lose the variation in land value inside a given municipality. We did the same for the income variable. As for the number of businesses and stores, as we are trying to measure the accessibility of resources and economic activity in a given geographical area, we assumed that using the total count per people in the municipality was an acceptable approximation (same value for each small area in the municipality), as we can assume that the businesses and stores inside the municipality are still relatively close (accessible) to every small areas inside this municipality.

**Table 2:** List of selected indicators for the social vulnerability analysis, with data source, spatial granularity and assumed direction towards vulnerability (i.e. plus sign means that the large the quantity, the larger the social vulnerability).

	Indicator	Data source	Spatial granularity	Direction towards vulnerability
1	Population density	eStat 2020 Japan national census	Small areas	+
2	Projected variation of pop. in 2050	Japan whole country small area level population evolution prediction system by Pr. Inoue, Aoyama university	Small areas	–
3	Part of residents below the age of 9	eStat 2020 Japan national census	Small areas	+
4	Part of residents above the age of 75	eStat 2020 Japan national census	Small areas	+
5	Part of residents that are women	eStat 2020 Japan national census	Small areas	+
6	Average real estate price (2021)	MLIT Land general information system	Municipalities	–
7	Real estate price evolution (2011-2021)	MLIT Land general information system	Municipalities	–
8	Average income (by cost of living)	eStat Statistical Observations of Municipalities 2022	Municipalities	–
9	Part of renting households	eStat 2020 Japan national census	Small areas	+
10	Number of businesses per people	eStat Statistical Observations of Municipalities 2022	Municipalities	–
11	Number of retail stores per people	eStat Statistical Observations of Municipalities 2022	Municipalities	–
12	Distance to closest medical facility	National land numerical data download service	Small areas	+
13	Distance to closest disaster shelter facility	National land numerical data download service	Small areas	+

**Figure 2:**
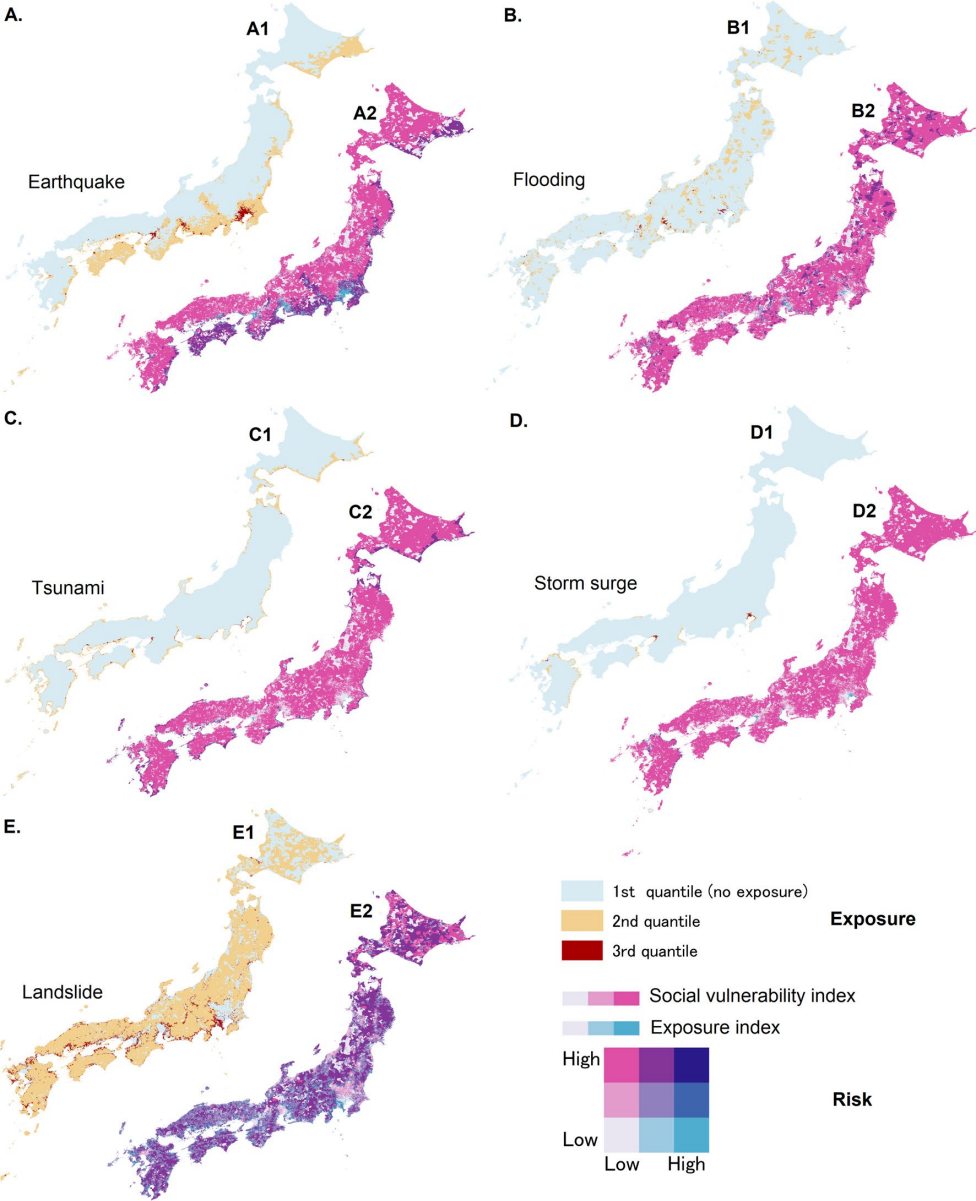
Measure of exposure (3 quantiles division) and visualisation of risk in a two-dimensional legend representation of social vulnerability and population exposure to 5 natural hazards in Japan. The left colour ramp (white/pink) represents 3 levels of social vulnerability while the right color ramp (white/light blue) represents 3 levels of exposure to earthquake disaster. Map generated using ArcGIS Pro 2.9.

To construct the social vulnerability index, the 13 indicators (Fig.2) in the input matrix were standardized to z-scores (0 means and unit standard deviation) to avoid large numbers that could bias the analysis. The Principal Component analysis (PCA) was performed (N = 120,000) in the software R, following the standard procedure. The PCA generated a new set of 13 orthogonal principal components. Then, by application of the Kaiser criterion (see Stafford 2017 study^17^ for a comparison of criterion used in similar studies), only the main components whose eigen value was superior or equal to 1 were selected (meaning that those are the components that explain more than a thirteenth of the total variance of the dataset). There were five remaining components.

**Figure 3:**
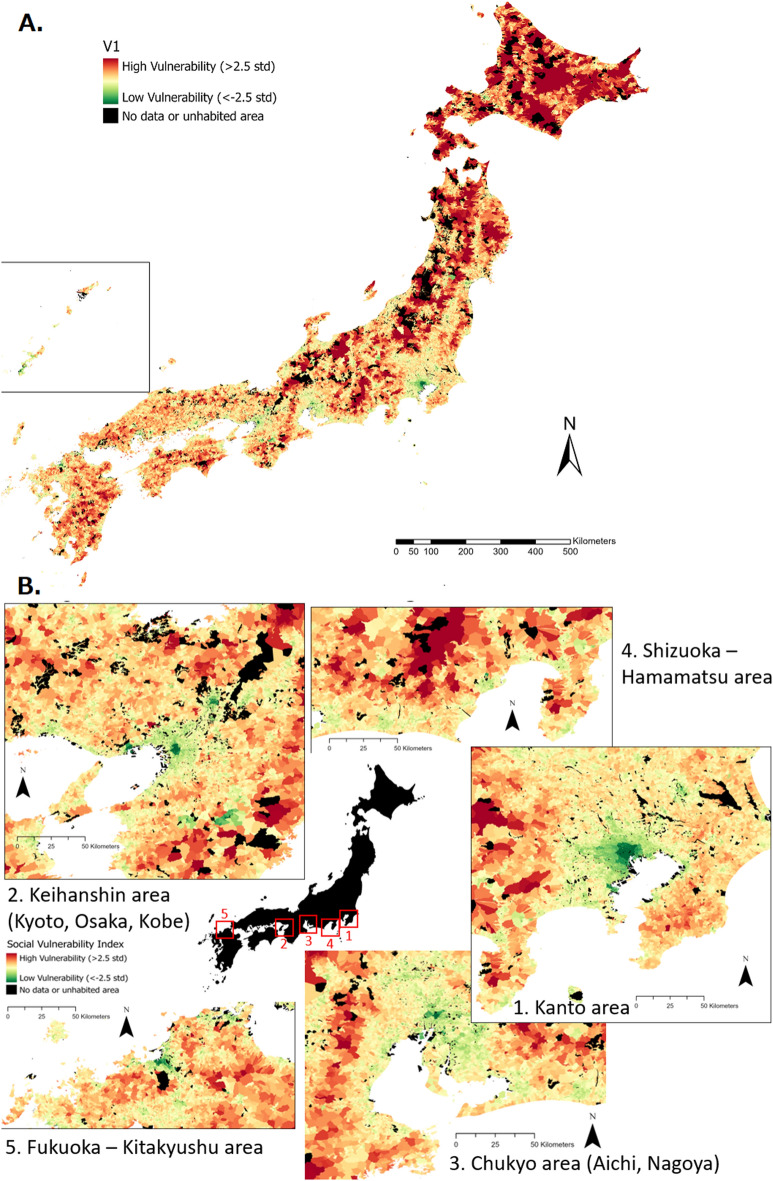
Measure of social vulnerability in Japan. (**A**) Whole country (**B**) Five major urban areas. The classification is made by standard deviation, where high vulnerability zones are red (more than 2.5 standard deviations above the average) and low vulnerability zones are green (more than 2.5 standard deviations below the average). Black zones are either unpopulated or lack data to be included in the analysis. Map generated using ArcGIS Pro 2.9.

In order to increase the interpretation of the five selected components and following the standard procedure, a Varimax rotation with Kaiser normalisation was performed with the software R, by rotating the axes of the components placing them as much apart from each other as possible. To facilitate the interpretation, loading values that are below 0.30 were suppressed. Based on these loadings (see Fig. 3), interpretation was done analysing what were the most important loading in each component and their sign. This helped to judge if the overall contribution of the component was increasing or decreasing social vulnerability, and attribute the appropriate sign, based on the experience of the authors. This interpretation is detailed in the Results section.

Finally, to obtain the final social vulnerability index, each component score was calculated, by computing the product of the loading with the indicator z-score value, then dividing by the number of indicators in this component, for every small zone (N = 120,000). Then, the five components are all summed, with their attributed sign, so that ultimately their contribution to social vulnerability is positive.

$$\begin{aligned} Component \, index= & {} \frac{\sum {loading*positive \, var}}{number \, of \, positive \, var} - \frac{\sum {loading*negative \, var}}{number \, of \, negative \, var}\\ Social \, vulnerability \, index= & {} \sum { component \, index} \end{aligned}$$

The social vulnerability index obtained for each small area was then z-score normalized again and imported inside ArcGIS Pro.

**Figure 4:**
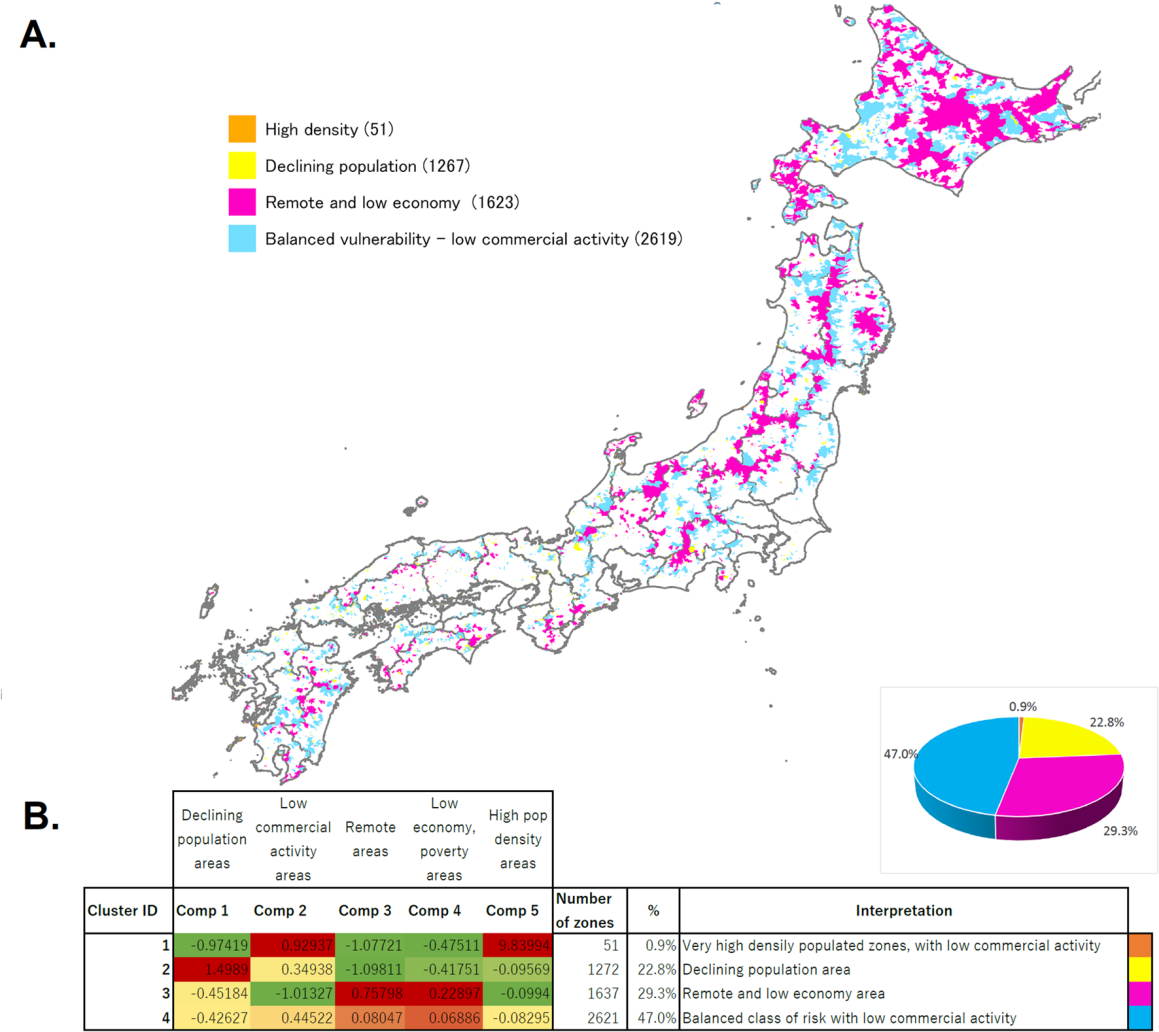
(**A**) Representation of the main patterns of high social vulnerability in Japan. (**B**) Result of clustering on high social vulnerability areas, with interpretation and distribution of the 4 clusters. Map generated using ArcGIS Pro 2.9.

In order to understand the different patterns of social vulnerability, the high social vulnerability zones were selected (social vulnerability index superior or equal to 1.5 standard deviation) and clustering was performed. The Kmeans algorithm was used with the ArcGIS function *Multivariate Clustering* and ran for K = 1 to K = 30. The K that maximises the pseudo-F statistic criteria is the number of clusters that best describe the dataset. Result showed a maximum pseudo-F with K = 4 clusters. The obtained clusters were then interpreted using the mean value of each component (see Fig. 4 and the “Results” section).

Ultimately, the results of the social vulnerability analysis and exposure analysis were compared in order to propose a representation of risk in Japan for five different types of disasters, with a very fine granularity.

### Data collection, treatments and representations

Data that were extracted for this analysis were available either for the prefecture level or smaller census level (municipalities and small areas). Each time, data layers merging had to be made to obtain the data on the full country scales. All Geographical Information Software (GIS) treatments were made using ArcGIS Pro 2.9. To obtain the distance from each small area to the small facilities, the Near function was used with reference to the barycenter of the small zone. Spatial joins were performed in order to join municipal level data to the small zone level data.

Regarding the construction of the exposure and risk matrix presented in the next chapter, this is how we proceeded. For the exposure maps, we did a spatial representation of the exposure indicator which calculation was explained in a previous paragraph, for each small areas of Japan. We divided all the small areas into 3 quantiles of exposure. The top quantile, which is the third of all small areas that are most exposed is shown in red. The same was done for the five considered natural hazards. It is worth mentioning that even if we call it exposure map, it is an indicator of exposure based on population data and the hazard map, as explained before in this “Methods” section.

For the risk maps, we combined the exact same exposure maps with the social vulnerability index that we have established. In the same way as the exposure maps, we divided the small areas into 3 quantiles of social vulnerability and then used a two dimensional legend to represent the risk. The high risk zones (dark purple), are the small areas which are simultaneously in the top quantile of exposure and in the top quantile of social vulnerability.

## Results

### Exposure analysis

The spatial representation of exposure to five different types of natural hazards is shown on Fig. 2A1–E1.

To represent the exposure on the maps in three level of intensity, we decided to divide all the small areas of Japan considered in the analysis into 3 quantiles based on the number of people exposed to the natural hazard in each small area. The first quantile has no or low exposure, the second quantile has intermediate exposure and the third one is the most exposed tier.

### Social vulnerability index

The principal component analysis was performed, and using the common Kaiser criterion, we only retain components that explain more than a thirteenth of the total variance (as we consider 13 social vulnerability variables). As a result, we have retained 5 components, accounting for 67.2% of the total variance. The Varimax rotation with Kaiser normalization insthen applied, and to ease the interpretation of each components, absolute loading values below 0.30 were suppressed.

**Table 3:** Rotated component matrix of the principal component analysis showing the indicator loadings and each component interpretation.

Input variable with direction towards vulnerability		Rotated components and loadings				
		1	2	3	4	5
+	Population density					− 0.99
−	Projected variation of population in 2050	0.53				
+	Part of residents below the age of 9	0.62				
+	Part of residents above the age of 75	− 0.86				
+	Part of residents that are women	− 0.61				
−	Average real estate price		− 0.45		0.79	
−	Average income (by cost of living)				0.8	
−	Real estate price evolution (2011-2021)				0.74	
+	Part of renting households	0.48		0.43		
−	Number of businesses in the muncipality		− 0.91		0.32	
−	Number of retail stores in the municipality		− 0.89			
+	Distance to closest medical facility			− 0.85		
+	Distance to closest shelter facility			− 0.84		
	Positive value loadings	Young population, renting		Renting	Real estate price, income, business	
	Negative value loadings	Fragile population	Real estate price, commercial activity	Distance to facilities		Population density
	Part of variance explained (total 67.2%)	15.9%	14.3%	13.4%	15.9%	7.7%
	Factor name (interpretation)	Population increase areas	Low commercial activity areas	Easy access to infrastruct.	High economy value areas	Low population density
	Direction for the vulnerability index	−	+	−	−	−
	Factor name after sign	Declining population areas	Low commercial activity areas	Remote areas	Low economy areas (poverty)	High population density

We can interpret the rotated component matrix Table 3 with the following five components of our vulnerability index. It is important to note that the names we are assigning the components of social vulnerability are a simplification that is the result of the authors interpretation of the indicators’ meanings and loadings. Moreover, this interpretation of variables can go beyond their simple meaning, in the sense that we are infering the meaning of the component based on the loadings of variables with regards to our experience. Consequently, this important step is open to discussion.

- *Declining population areas*: there is an emphasis on the portion of elders, while the portion of children and population growth are also emphasized by the loadings with an opposite sign. Zones where the young population is important, with a positive variation of population expected, whereas the population of elders is on an opposite trend is interpreted as population dynamism areas, which in we see as a factor reducing the vulnerability, thus we are deciding to put a minus sign. After signing, this component represents ”Declining population areas”.
- *Low commercial activity areas*: the component value is high when the number of businesses and retail store is low in the municipality, as well as a low real estate price. A tendency in low prices and number of businesses suggests us a low commercial activity of these areas.
- *Remote areas*: the component value is high when the distance the distance to the closest hospital and disaster shelter is high. Naturally, this components emphasises the areas that are remote from these infrastructures.
- *Low economy, poverty areas*: we notice that this component’s value is high when real estate prices and income are high. This means that this component emphasizes high economy value areas. This reduces the social vulnerability to us, so we assign a minus sign and the component becomes ”low economy areas”.
- *High population density areas*: this component only includes the population density indicator.

#### Social vulnerability results

For each component, an index was calculated with the respective loadings of each variables, and then the five index were summed with the interpreted sign in the Varimax matrix Table 3 to obtain the final social vulnerability index. More details are provided in “Methods”.

The spatial representation of the social vulnerability index for the whole country (A) and for 5 major urban areas (B) are shown in Fig. 3.

To represent the social vulnerability on the maps, we represented the index after Kaiser normalization, and the scale goes from dark green (very low vulnerability, more than 2.5 standard deviations below the average of the small areas), to yellow (average of social vulnerability index in all small areas) to dark red (very high social vulnerability, more than 2.5 standard deviations above the average of the small areas).

We notice larger zones of social vulnerability in the northern part of Japan, especially the Tohoku and Hokkaido regions. We also notice a pattern of very low vulnerability in the center of major cities, which is not surprising as they are areas of wealth and infrastructures concentration. Japan country side areas are well known to suffer heavily from depopulation and aging of population^19^, making these areas vulnerable. These aspects were well captured by this index.

#### Different patterns of social vulnerability

High social vulnerability areas show different vulnerability profiles depending on which components are more prominent. We decided to apply a clustering algorithm in order to understand the main patterns of high social vulnerability areas in Japan. The Kmeans clustering algorithm was applied only to high social vulnerability areas (standard deviation of 1.5 and above), and best fit for the data was reached with K = 4 clusters. A map of the clustered social vulnerability patterns is shown in Fig. 4A and cluster interpretations can be view in Fig. 4B. The 4 clusters can be interpreted as follows (from highest to lowest cluster count):

- *Balanced vulnerability*: low commercial activity vulnerable areas: zones where all components are vulnerable, with especially a low number of retail and businesses.
- *Remote and low economy vulnerable areas*: zones that have very low access to hospitals or shelters as they are situated in remote areas (such as mountain areas) combined with low land value and income.
- *Declining population vulnerable areas*: zones where aging of population is severe.
- *High density vulnerable areas*: zones (exception, only 51) where density of population is high but economical and social wealth is not high as in large cities.

Pink zones show that remoteness is the main contributing factor of vulnerability in many zones in northern Japan. The blue zones, which show a vulnerability distributed on low commercial activity, low economy and remoteness, seem to be spread all across the country. Declining population vulnerable areas, which are more scarce, seem to be situated in peripheral zones of medium size cities.

### Picture of risk in Japan

**Figure 5:**
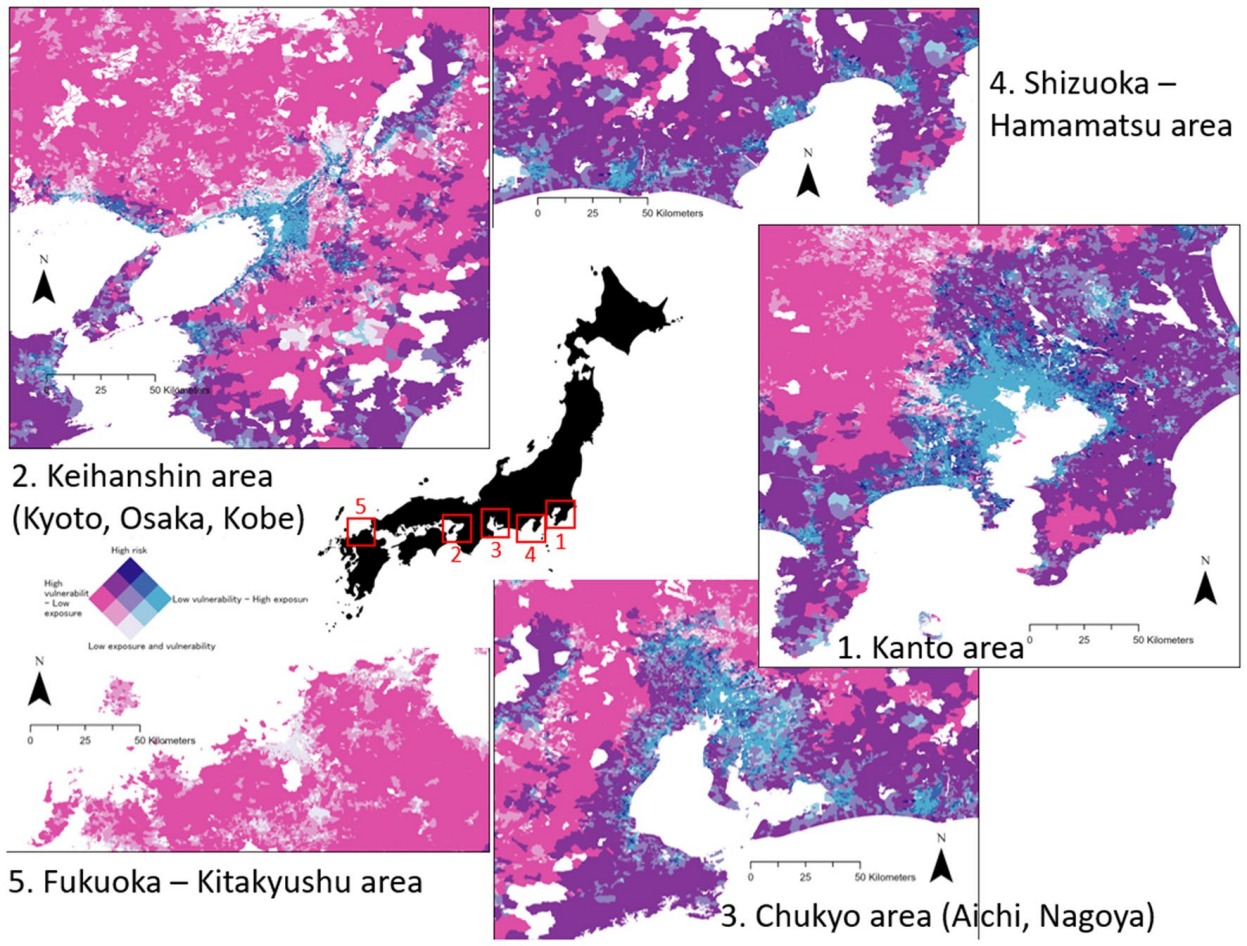
Earthquake risk in major urban areas in Japan (ranking by order of importance), two-dimensional legend representation of social vulnerability and population exposure to earthquake disaster. The left colour ramp (white/pink) represents 3 levels of social vulnerability while the right color ramp (white/light blue) represents 3 levels of exposure to earthquake disaster. The focus is on the high vulnerability areas with high exposure to earthquake hazard, resulting is high risk area for earthquake disaster. Map generated using ArcGIS Pro 2.9.

The spatial representation of risk analysis is shown on Fig. 2A2–E2. The representation of risk to earthquake hazard zoomed out on five main urban areas in Japan is shown on Fig. 5.

In order to visualise the high risk zones for each natural hazard, we used the bivariate representation tool of ArcGIS. The first variable to be represented is the social vulnerability index, and the second one is the density of exposed population. Both indicators are divided into three quantiles (low, medium and high) of equal number of features. The high risk zones are the ones that simultaneously have a highest quantile social vulnerability and a highest quantile density of exposed population.

As an example, regarding earthquake hazard, our obtained data results about earthquake disaster risk show us that, out of about 75,500,000 individuals exposed to the high intensity shaking in the next 30 years in Japan, about 2,875,000 individuals are residents of one of the 3460 high risk zones (both high social vulnerability and high exposure to this hazard).

## Discussion

In this study, a simple method to measure the exposure of populations to five different types of natural hazards was established. Naturally, it showed that high exposure zones are totally different for different types of hazards. Regarding the earthquake hazard, we notice that the exposure is mainly focused on high density urban areas of Japan (Tokyo, Kanagawa and Chiba with the threat of inland earthquake under the Tokyo bay and Shizuoka, Nagoya and Osaka with the threat of Nankai trough inter-plate earthquake). Overall, the entire west coast of Japan from northern Hokkaido to southern Kyushu is exposed to earthquake hazard.

We have established the first Japan countrywide social vulnerability index with a fine spatial resolution, with the help of principal component analysis and 13 indicators constructed from various open data sources. The method followed for the construction of this index is within the common methods for social vulnerability estimation, adapting the indicators to the Japanese context considering data availability, support of the indicators by the literature and balance of the indicators to represent various aspect of vulnerability. Our social vulnerability index is derived from 5 components representing key aspects of social vulnerability to natural hazards: declining population, low commercial activity, remoteness, low economic activity, high population density.

When taking a wide look at the country-wide spatial distribution of social vulnerability Fig. 3A, it appears that social vulnerability is highly heterogeneous, and two notable observations can be made:

- An urban/rural fracture: the largest urban centers of the country have the lowest vulnerability index values, whereas more rural regions seem to have the highest social vulnerability. This is coherent with what similar studies have observed, for instance in Australia^5^.
- A north/south fracture: high social vulnerability seems to be more spatially prominent in the north part of Japan, especially in Hokkaido and the Tohoku region.

If we look more closely at social vulnerability in the main urban areas of Japan Fig. 3B, it appears that the lowest vulnerability match with the main economic centers of the country (central Tokyo, Osaka or Kyoto). The more distance from the centers, the higher the vulnerability. Outskirts of the cities can either have average or high vulnerability.

By applying the proposed method, we have suggested a way to represent the risk associated with each type of natural hazard in Japan. This double scale mapping allows us to easily visualise which zones are characterized by high social vulnerability and are very exposed to a certain type of hazard.

As we can observe on Fig. 5 with the example of risk to earthquake in major urban areas of Japan, there seems to be a pattern in the spatial distribution of high risk areas. As we explained earlier, large city centers show the lowest social vulnerability values, which tends to become higher in the suburban and rural areas. but are coincidentally also the highest exposure zones facing earthquakes. This leads to the high risk areas being situated on the urban fringes of the large agglomerations, where many people are exposed to the earthquake hazard but the access to facilities and social capital of populations is reduced, making these areas socially vulnerable.

Risk to landslide (Fig. 2E2) is spread across the country, as the exposure to landslide is particularly spread out compared to other natural hazards. An interesting example to notice is the concentration of high to medium risk to landslide in the Kanagawa prefecture, especially on the Shonan zone and Miura peninsula. It is a highly populated area, and the zone is being under the imminent threat of a south Kanto earthquake^21^ that would trigger strong motion leading to landslides in the area (which has actually happened before in the 1923 Great Kanto earthquake)^22^.

The approach developed in this study helps to reveal the large variety of risk zones in Japan. This process can be easily replicated as all data are freely accessible. It should be seen as a decision-making tool for policy makers and local leaders that are responsible for local disaster mitigation measures implementation and urban planning, as it ranks the areas from most to less risky. Of course, the criteria for such a ranking can be adapted to the relevant context: which natural hazards to consider, which administrative scale (small areas, district, municipalities...), which components of social vulnerability should be more emphasized, for example. Hopefully, this work will contribute to raise policy-makers attention towards the fact that riskier areas and the associated populations are not always the most exposed to natural disasters. It is also noteworthy that the general public can make a good use of these data, especially spatial representations of risk on maps, and obtain a better understanding of how vulnerable is their living community overall and to which kind of risk they are most exposed.

However, it is important to mention the limitations in this work. Firstly, the inherent limitations of social vulnerability assessment with statistical methods, such as consistency issues and the importance of variable selection^23^ where different experts will often have diverging opinions. Secondly, we shall mention the limitations of the data for exposure assessment, that are based on government and prefecture level scenarios (all possible scenarios cannot be anticipated, and assumptions for the disaster scenarios can always be discussed). Thirdly, there are factors that might not have been taken into account because open data availability is still limited, and we hope to be able to refine the analysis when more data of better quality will be made accessible, by for example adding more natural disasters (such as volcano eruptions, liquefaction zones after earthquakes), more factors of vulnerability, such as the physical vulnerability of the buildings (structure or material) or factors related to individual coping capacity (household losses insurances for instance). Fourthly, our study noticed a clear pattern of low vulnerability in large cities (areas of asset concentration). This means that people living in these areas tend to have higher support capabilities to face the consequences of disasters, and are expected to have a better recovery. It doesn’t mean that the economic or human loss would not be high in these areas in case of disaster, as large cities typically have a high concentration of assets, and high population density. Therefore, it is important to keep in mind that this kind of study did not measure the potential economic loss due to disaster, but uses indicators as proxies to estimate how population can support themselves or not facing disastrous events. Lastly, in the future more effort should be put into the development of independent secondary datasets that could be used to validate social vulnerability and exposure indexes^24^. This has been done in the past in other regions of the world such as Germany^7^, but remains difficult as broad concept like social vulnerability cannot be directly measured, and precise surveys of social impacts after the occurrence of disasters are needed, which require important means, preparation and reactivity.

## Conclusions

This paper has developed and successfully performed a methodology, using only publicly available data to create the first large-scale (whole country), fine resolution (sub-municipal level) social vulnerability, exposure and risk analysis of Japan, in the context of five different types of natural hazards (earthquake, tsunami, storm surge, flooding and landslide).

Patterns of social vulnerability in Japan were represented and analyzed, revealing heterogeneity with urban/rural and north/south fractures. Social vulnerability is the lowest within the main economic centers of the country (Tokyo, Osaka, Kyoto for example), whereas rural areas have a high social vulnerability. Cities outskirts have an average to high vulnerability.

High population exposure zone distributions are totally different depending on the natural hazard considered. In the case of earthquakes, the exposure is focused mainly in high density urban areas, located on the west coast.

The approach developed in this study has revealed a large variety of risk zones in Japan. High risk areas to earthquake disaster are situated on the urban fringes of large agglomerations, where populations are exposed to the earthquake hazard but lack social capital and access to facilities. The east part of Kanagawa prefecture is under a high risk of landslide hazard, which is concerning as a strong Kanto inland earthquake is predicted in the coming years, which could trigger important landslides in this highly populated area.

As this process can be easily replicated, using only public open data, we hope that this research will inspire the development of similar indexes and decision making tools to identify the highest risk areas in Japan. We believe such tools are needed and useful for policy-makers as well as the general public.

## Data Availability

All data used in this study are publicly available, and the sources are detailed below. The data used to build the indicator of social vulnerability come from the following sources. The small zone mesh that is the base mesh of the analysis was retrieved from eStat, Japan national census, boundary data section, available here: www.e-stat.go.jp. Population density, part of residents below the age of 9, part of residents above the age of 75, part of residents that are women, part of renting households were derived from data retrieved in the 2020 Japan national census, available here: www.e-stat.go.jp. The projected variation of population in 2050 was extracted from the Japan whole country small area level population evolution prediction system by Professor Inoue from Aoyama university, available here: agu-econ.maps.arcgis.com. The average real estate price in 2021 and evolution of real estate price from 2011 to 2021 were derived from data retrieved in the Land general information system database from the Ministry of Land, Infrastructure and Transport, available here: www.land.mlit.go.jp. Average income, number of businesses and number of retail stores were derived from data from eStat in the Statistical Observations of Municipalities 2022 section, available here: www.e-stat.go.jp. Cost of living was retrieved from eStat in the Statistical Observations of Prefectures 2022 section, available here: www.e-stat.go.jp. Geographical data about medical facilities and shelter facilities were retrieved from the National land numerical data download service, from the Ministry of Land, Infrastructure and Transport, available here: nlftp.mlit.go.jp. The data used to build the exposure indicator to different hazards come from the following sources. Probabilistic seismic data was retrieved from the Japan Seismic Hazard Information Station, available here: www.j-shis.bosai.go.jp. Hazard zones for tsunami, storm surge, river flooding and landslide hazards were retrieved from the National land numerical data download service by the Ministry of Land, Infrastructure and Transport, available here: nlftp.mlit.go.jp. The 500m grid mesh was retrieved from eStat, Japan national census, boundary data section, available here: www.e-stat.go.jp. The population data on this grid was retrieved from eStat, 2020 Japan national census, available here: www.e-stat.go.jp.
